# The Aqueous Extract of *Ficus religiosa* Induces Cell Cycle Arrest in Human Cervical Cancer Cell Lines SiHa (HPV-16 Positive) and Apoptosis in HeLa (HPV-18 Positive)

**DOI:** 10.1371/journal.pone.0070127

**Published:** 2013-07-26

**Authors:** Amit S. Choudhari, Snehal A. Suryavanshi, Ruchika Kaul-Ghanekar

**Affiliations:** Cell and Translational Research Laboratory, Interactive Research School for Health Affairs (IRSHA), Bharati Vidyapeeth University Medical College Campus, Dhankawadi, Pune, India; Albany Medical College, United States of America

## Abstract

Natural products are being extensively explored for their potential to prevent as well as treat cancer due to their ability to target multiple molecular pathways. *Ficus religiosa* has been shown to exert diverse biological activities including apoptosis in breast cancer cell lines. In the present study, we report the anti-neoplastic potential of aqueous extract of *F. religiosa* (FR_aq_) bark in human cervical cancer cell lines, SiHa and HeLa. FR_aq_ altered the growth kinetics of SiHa (HPV-16 positive) and HeLa (HPV-18 positive) cells in a dose-dependent manner. It blocked the cell cycle progression at G_1_/S phase in SiHa that was characterized by an increase in the expression of p53, p21 and pRb proteins with a simultaneous decrease in the expression of phospho Rb (ppRb) protein. On the other hand, in HeLa, FR_aq_ induced apoptosis through an increase in intracellular Ca^2+^ leading to loss of mitochondrial membrane potential, release of cytochrome-c and increase in the expression of caspase-3. Moreover, FR_aq_ reduced the migration as well as invasion capability of both the cervical cancer cell lines accompanied with downregulation of MMP-2 and Her-2 expression. Interestingly, FR_aq_ reduced the expression of viral oncoproteins E6 and E7 in both the cervical cancer cell lines. All these data suggest that *F. religiosa* could be explored for its chemopreventive potential in cervical cancer.

## Introduction

Cervical cancer is the second major cause of cancer death in the women all over the world [1], [2]. High-risk human papilloma viruses (HPVs) such as HPV 16, 18, 31 and 33 have been attributed to be the major risk factors for cervical cancer, out of which HPV-16 and -18 account for almost 70% of the cancers [3]. E6 and E7 are the two viral oncoproteins necessary for the development and maintenance of the transformed phenotype in cervical cancer cells. E6 promotes p53 degradation through a ubiquitin-dependent proteasome pathway while E7 associates with retinoblastoma (pRb) protein and interferes with its binding to E2F [4], [5]. This results into loss of Rb/E2F complexes leading to release of transcription factor E2F that induces the expression of cell proliferative genes [5].

Although the current treatment modalities can cure 80–95% of early-stage and 60% of loco-regionally advanced cancers, the recurrent and metastatic disease still remains a major problem [6]. Recently, Complementary and Alternative Medicine (CAM) is gaining popularity as a chemopreventive approach towards the management as well as prevention of cancer recurrence [7], [8]. More than 60% of currently used anti-cancer drugs are originally derived from natural sources such as plants, marine organisms and microorganisms [9]. Various scientific studies, including ours, have suggested the potential of medicinal plants as anti-cancer drug candidates [10], [11]. We have recently reported the anticancer potential of *Cinnamomum cassia* (cinnamon) in cervical cancer [12].

*Ficus religiosa* L. family Lauraceae, has been extensively used in the traditional medicine for various disorders. Its different parts have been used medicinally in different forms as well as in combination with other herbs [13], [14]. It has been shown to exhibit diverse biological activities [14] including wound healing [15], anti-bacterial [16], anti-convulsant [17], anti-diabetic [18], [19], anti-inflammatory [20], acetyl cholinesterase inhibitory activity [21] and anti-anxiety activity [22]. The acetone extract of *F. religiosa* leaves has been shown to induce apoptosis in breast cancer cell lines [14].

We have recently reported the antioxidant and cytotoxic activity of *F. religiosa* bark in cervical cancer cells [23]. In the present study, we have investigated the putative molecular mechanism underlying the antineoplastic potential of the aqueous extract of *F. religiosa* (FR_aq_) bark in cervical cancer. Our data suggests that Ficus inhibits the growth of cervical cancer cell lines, SiHa and HeLa, by inducing cell cycle arrest and apoptosis, respectively. Interestingly, FR_aq_ significantly reduced the expression of viral oncoproteins E6 and E7, thereby suggesting the therapeutic potential of *F. religiosa* in cervical cancer.

## Materials and Methods

### Chemicals and Reagents

Tissue culture plasticware was purchased from BD Biosciences (CA, USA) and Axygen Scientific Inc (CA, USA). Dulbecco’s Modified Eagles Medium (DMEM) powder, penicillin and streptomycin were obtained from Invitrogen/Gibco (Grand Island, NY, USA). Fetal bovine serum (FBS), 3-(4,5-dimethylthiazol-2-yl)-2,5-diphenylthiazolium bromide (MTT), FCCP, Ionomycin and JC-1 were purchased from Sigma-Aldrich (St. Louis, MO). Primary antibody against p53 (DO-1), p21 (187), caspase-3 (H-277), cyto-c (7H8), Her-2 (F-11), pRb (C-15), ppRb (SER 807/811), HPV16 E6/18 E6 (C1P5), HPV16 E7 (ED17), HPV18 E7 (N-19) or tubulin (B-7) were purchased from Santa Cruz Biotechnology, Inc. (Santa Cruz, CA, USA). Annexin V-FITC apoptosis kit #3 was purchased from Invitrogen (CA, USA). All other common reagents were procured from Qualigens Fine Chemicals (Mumbai, India).

### Preparation of Aqueous Extract of *Ficus religiosa* (FR_aq_) and Preliminary Phytochemical Investigations

The bark of Ficus *religiosa* L. was collected from Pune District, Maharashtra, India and it was authenticated as described previously [23]. The voucher specimen (MPCC 2417) of authentic plant species was deposited at the herbarium of Medicinal Plants Conservation Center (MPCC), Pune, Maharashtra, India. The bark was weighed, powdered and extracted in double distilled water in a hot water extractor as described previously [23], [24]. The resulting extract was centrifuged at 13000 rpm for 15 min to remove the particulate matter. The supernatant was further filter-sterilized using Swiney filter (pore size, 0.45 µm) and the resultant filtrate was stored in aliquots at −80°C until use. The yield of the dried extract obtained from the starting crude material was 8.6% (w/w). The freshly prepared FR_aq_ extract was qualitatively tested for the presence of flavonoids, phenols, saponins, tannins and carbohydrates using standard procedures of analysis [25].

### Cell Culture

The human cervical carcinoma cell lines, SiHa (HPV-16), HeLa (HPV-18) and C33A (HPV-negative) were obtained from National Center for Cell Science (NCCS), Pune, Maharashtra, India. The cells were grown in DMEM supplemented with 10% FBS, 2 mM L-glutamine, and antibiotics (100 units/ml penicillin and streptomycin). The cells were incubated in a humidified 5% CO_2_ incubator at 37°C.

### Cell Growth Analysis

The assay was performed as described previously [12]. Briefly, SiHa and HeLa cells were seeded at a density of 1×10^5^ and 1.5×10^5^ cells/ml, respectively, in 24-well plates in triplicates. Next day, the cells were treated with different concentrations of FR_aq_ (0–80 µg/ml) for 24, 48 and 72 h. The cells were harvested and counted for viability using trypan blue dye exclusion method [12], [26].

### Cell Growth in Monolayer

The assay was performed as described previously [12]. Briefly, SiHa and HeLa cells were plated at a seeding density of 1×10^3^ cells/ml in 6-well plates. After 24 h, the cells were exposed to various concentrations of FR_aq_ (0–80 µg/ml) followed by incubation at 37°C in a 5% CO_2_ incubator for one week in presence of the extract. This was followed by fixing the colonies with 4% paraformaldehyde and staining with 0.5% crystal violet. The colonies were photographed with Sony DSC-S75 Cyber-shot camera.

### Cell Growth in Soft Agar Assay

The assay was performed as described previously [12], [26]. Briefly, SiHa and HeLa cells (5×10^3^ cells/ml) along with different concentrations of FR_aq_ (0–80 µg/ml) were mixed with 0.35% agarose (DNA grade, GIBCO BRL) in complete DMEM medium at 40°C and gelled at room temperature for 20 min over a previously gelled layer of 0.5% agarose in complete medium in 6-well plates. After incubation for two weeks, the colonies were counted in 10 different fields using an Axiovert 200 M microscope (Carl Zeiss, Germany) and the average value was calculated.

### Wound Healing Assay

Both SiHa and HeLa cells were seeded at a density of 4×10^5^/ml in 24-well plates and were allowed to adhere overnight at 37°C in 5% CO_2_ incubator. Next day, the cells were starved for serum for 6 h, followed by addition of complete medium with or without FR_aq_ (0–80 µg/ml). An artificial wound was made in the plates containing treated and untreated cells with a 10µl micropipette tip and the wound was allowed to heal for 24 h at 37°C in 5% CO_2_ incubator. The images for 0 h as well as 16 h were captured with the help of Axiovert 200 M microscope. The average extent of wound closure was evaluated by measuring the width of the wound by using ImageJ 1.44p [27].

### Matrigel Transmembrane Invasion Assay

For invasion studies, 24-well BioCoat Matrigel Invasion Chambers (BD Bioscience, Bedford, MA) were used [28]. SiHa and HeLa cells (5×10^4^) with or without FR_aq_ treatment (0–80 µg/ml) were seeded in serum-free medium into the upper invasion chambers and allowed to invade across the Matrigel-coated membrane for 24 h. The medium containing 10% FBS was added to the lower chamber which served as a chemo attractant. After 24 h of incubation, non-invading cells were removed from the top of each membrane with wet cotton swabs; invading cells attached to the bottom of the membrane were fixed with 4% formalin and stained using 0.5% crystal violet. The cell numbers were counted in ten random high-power (×20) fields using Axiovert 200 M microscope (Carl Zeiss, Germany) equipped with a Sony Cyber-shot 3.3 mega pixels camera.

### Gelatin Zymography

The activity of MMP-2 in the conditioned medium was determined by gelatin zymography as described previously [12]. Briefly, SiHa and HeLa cells were seeded at a density of 4×10^5^ cells/ml in 6-well plates and allowed to adhere overnight at 37°C in 5% CO_2_ incubator. Next day, the cells were treated with various concentrations of FR_aq_ (0–80 µg/ml) prepared in serum-free medium and incubated for 24 h. The following day, the culture medium was collected and centrifuged at 14,000 rpm for 20 min at 4°C to remove the cellular debris. The conditioned medium from control cells as well as cells treated with FR_aq_ was collected and concentrated in Centricon YM-30 tubes (Millipore, MA). The samples containing an equal amount of total proteins, were mixed with sample buffer (2% SDS, 25% glycerol, 0.1% bromophenol blue and 60 mM Tris-HCl pH 6.8) and subjected under non-reducing conditions on to 7.5% SDS-polyacrylamide gel containing gelatin (0.5 mg/ml). Following electrophoresis, the gel was washed with 0.25% Triton X-100 and incubated over night at 37°C in buffer containing 150 mM NaCl, 100 mM CaCl_2_, 50 mM Tris-HCl pH 7.5, 1% Triton X- 100, 0.02% NaN_3_. The gel was stained with staining solution (0.1% Coomassie Brilliant blue R-250 in 40% isopropanol) and destained in 7% acetic acid. Gelatinolytic activity was detected as unstained bands against blue background. The quantification of bands in control and treated samples was performed by densitometric analysis on Alpha Imager using Alpha Ease FC software, Alpha Innotech.

### Immunoblotting

HeLa and SiHa cells were plated at a seeding density of 4×10^5^ cells/ml in 6-well plates and allowed to adhere overnight at 37°C in CO_2_ incubator. Next day, the cells were exposed to various concentrations of FR_aq_ (0–80 µg/ml) and incubated for 24 h. Following incubation, the cells were harvested by trypsinization, washed with 1×PBS and protein was extracted as described previously [12]. Briefly, the cell pellets were resuspended in 60 µl lysis buffer containing 50 mMTris (pH 7.4), 5 mM EDTA, 0.5% NP40, 50 mM NaF, 1 mM DTT, 0.1 mM PMSF, 0.5 µg/ml leupeptin (Pro-pure Amersco, Solon, USA), 1µg/ml pepstatin (Amresco, Solon, USA), 150 mM NaCl, 0.5 µg/ml aprotinin (Amersco, Solon, USA) and protease inhibitor cocktail (Roche, Lewes, UK) and incubated on ice for 1 h with intermittent mixing. The extract was centrifuged for 20 min at 4°C at 12000 rpm. For cytochrome-c release, cytosolic and mitochondrial fractions were prepared as described previously [29]. The protein was estimated using Bradford reagent (Biorad Laboratories Inc, CA, USA). Equal amount of protein was loaded on to either 10% or 12% (for E6 protein) SDS-polyacrylamide gel and transferred electrophoretically to Amersham Hybond-P PVDF membrane (GE Healthcare, UK) in sodium phosphate buffer (pH 6.8). The membrane was blocked in 5% BSA in TST and incubated at 4°C overnight with primary antibody against p53, p21, caspase-3, cyto-c, Her-2, pRb, ppRb, HPV16 E6/18 E6, HPV16 E7, HPV18 E7 or tubulin (Santacruz, CA, USA) at a 1∶500 dilution. The membrane was washed in TST and incubated with secondary IgG HRP conjugate at 1∶5000 dilution. Proteins were visualized with a chemiluminescence kit (Amersham ECL Advance western blotting detection kit, GE Healthcare, UK) and densitometry analysis was performed on scanned immunoblot images using the Image J gel analysis tool.

### Assessment of Cell Cycle Arrest

For cell cycle analysis, HeLa, SiHa and C33A cell lines were plated at a seeding density of 5×10^5^ cells/well in 6-well plates and allowed to adhere for 24 h at 37°C in CO_2_ incubator. Next day, the cells were treated with FR_aq_ (0–80 µg/ml) for 24 h. The cells were harvested by trypsinization and fixed in ice-cold 70% ethanol at −20°C for 30 min. Following washing with 1×PBS, the cells were treated with RNAse A (100 mg/ml) at room temperature for 30 min and stained with PI (20 µg/ml). Stained cells were analyzed for DNA-PI fluorescence using a flowcytometer (FACS Calibur, BD). A minimum of 10,000 events were counted per sample; data were analyzed using FACS Calibur-cell quest software (Becton Dickinson) for the proportions of cells in G_0_/G_1_, S phase and G_2_/M phases of the cell cycle.

### Assessment of Apoptosis

To determine the number of cells undergoing apoptosis upon FR_aq_ treatment, HeLa, SiHa and C33A were plated at a seeding density of 5×10^5^ cells/well in 6-well plates and allowed to grow overnight at 37°C in CO_2_ incubator. Next day, the cells were treated with various concentrations of FR_aq_ (0–80 µg/ml) and incubated for 24 h. Cells were stained with Annexin V-FITC according to manufacturer's instructions (Annexin V-FITC apoptosis kit #3, Invitrogen, Grand Island, NY). A total of 10,000 events were acquired and dual parameter dot plot of FL2-H (X-axis; PI fluorescence, linear scale) versus FL1-H (Y-axis; Annexin V-FITC-fluorescence, linearscale) was recorded. The data was analyzed using the FACS CaliburCell Quest software (Becton Dickinson).

### Analysis of Mitochondrial Membrane Potential (Δψm)

Flow cytometry analysis was performed on cells using JC-1 dye as described previously [12]. HeLa cells were plated at a seeding density of 5×10^5^ cells/well in 6-well plates and allowed to adhere overnight at 37°C in CO_2_ incubator. Next day, the cells were treated with FR_aq_ (0–80 µg/ml) for 24 h. This was followed by harvesting the cells, washing twice with 1× PBS followed by incubation with fresh culture media containing JC-1 dye (2.5 µg/ml) for 30 min at 37°C in dark. Stained cells were washed twice with ice-cold 1×PBS, re-suspended in 1 ml 1×PBS and analyzed for Δψ*m* by flow cytometry. FCCP (10 µM) was used as a positive control. A minimum of 10,000 events were counted per sample and the fluorescence intensities were measured at 527 nm (green) and 590 nm (red).

### Detection of Intracellular Calcium using Fluo-3/AM

HeLa cells were plated at a seeding density of 5×10^5^ cells/well in 6-well plate and allowed to adhere overnight. Next day, the cells were treated FR_aq_ (0–80 µg/ml) for 24 h at 37°C in 5% CO_2_. Following the incubation, intracellular Ca^2+^ levels were analyzed by flow cytometry as described previously [12]. Briefly, the cells were loaded with 5 µM Fluo-3/AM (Sigma, St. Louis, MO) and 100 µg/ml of Pluronic F127 (Sigma, St. Louis, MO) in centrifuge tubes and incubated at 37°C, 5% CO_2_ for 1 h in the dark. The cells were resuspended after every 20 min to ensure even dye loading. The cell pellets were washed twice with 0.9% saline and resuspended in 3 ml Hank’s Balanced Salt Solution (HBSS) in FACS tubes. Ionomycin (30 µM) was used as a positive control. Fluorescence intensities were measured at 525 nm by FACS Calibur (Becton Dickinson Immunocytometry Systems, San Jose, CA) to obtain baseline readings. Mean channel fluorescence intensities were calculated using CellQuest software.

### Statistical Analysis

All the experiments were performed in triplicates and repeated at least three times and the data has been presented as mean ± SD. Statistical analysis was conducted with the SigmaStat 3.5 program (Systat Software, Inc.) using one-way ANOVA with α = 0.05.

## Results

### Ficus Modulates the Growth Kinetics of Cervical Cancer Cells

**Figure 1:**
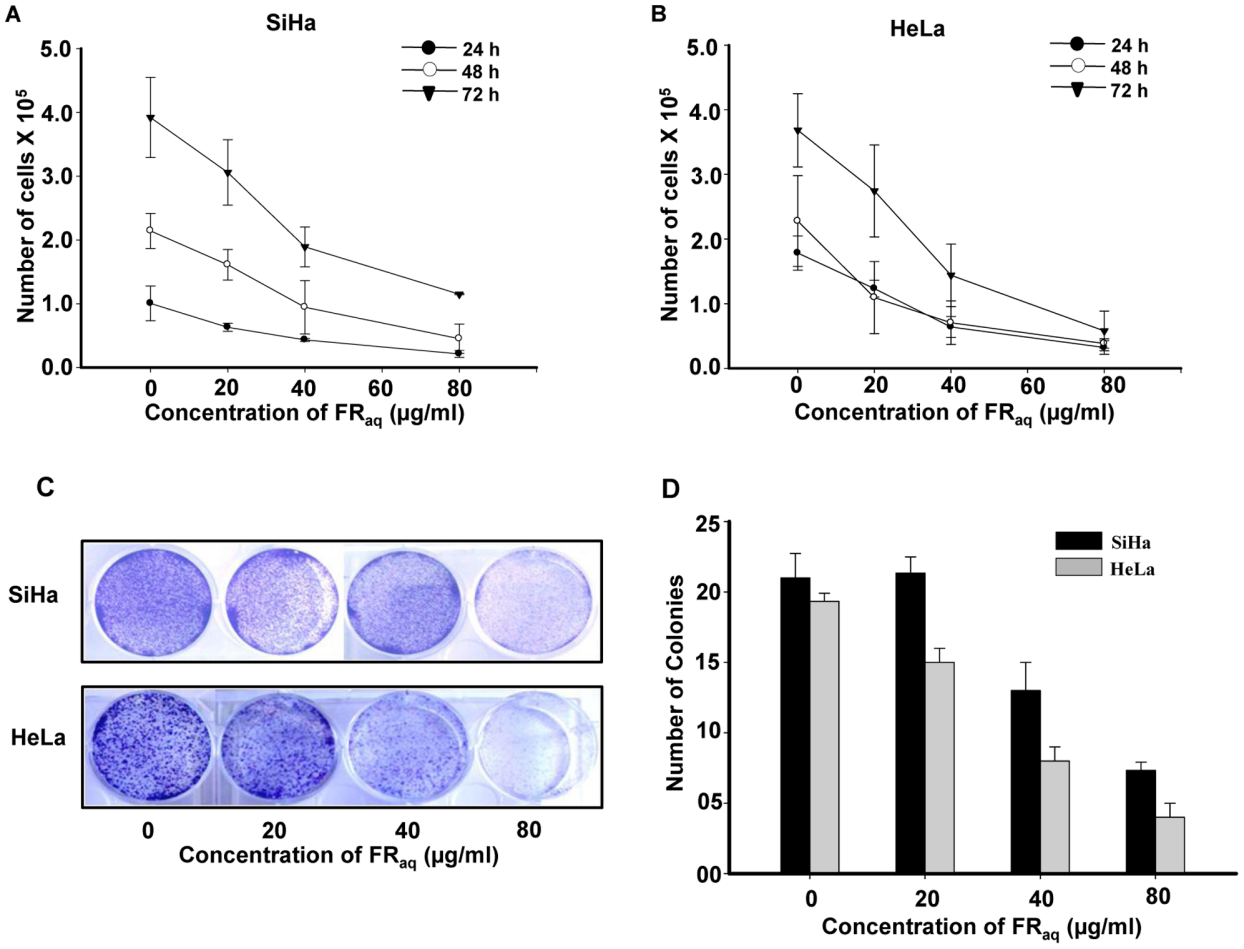
Ficus regulates the growth of cervical cancer cells. SiHa (**A**) and HeLa (**B**) were treated with FR**_aq_**(0–80 µg/ml) for 24–72 h and the number of viable cells were counted using the trypan blue dye exclusion method. Data represent mean ± SD of three independent experiments. (**C**) The cervical cancer cell lines (SiHa and HeLa) were treated with FR**_aq_**(0–80 µg/ml) for one week. The colonies were stained with crystal violet and photographed. The experiments were repeated three times. (**D**) Both SiHa and HeLa (5×10^3^) along with FR**_aq_** (0–80 µg/ml) were grown in soft agar for two weeks. Colonies were counted from at least 10 different areas and the average of each has been plotted. The data represents mean ± SD of five independent experiments.

We have previously reported that *F. religiosa* exhibited significant antioxidant potential as well as cytotoxicity in cervical cancer cell lines, HeLa and SiHa [22]. Based on it, we chose non-cytotoxic concentrations of FR_aq_ (0–80 µg/ml) for SiHa (HPV-16) and HeLa (HPV-18) in our assays. It was observed that FR_aq_ decreased the growth of the cells in a dose- and time-dependent manner. In SiHa, FR_aq_ decreased the cell growth at 80 µg/ml concentration by ∼4.78−(p = 0.008), ∼4.72−(p = 0.001) and ∼3.42-fold (p = 0.053) at 24, 48 and 72 h, respectively, compared to the untreated control cells (Figure 1A). Similarly, at 80 µg/ml concentration of FR_aq_, HeLa cells exhibited ∼5.53−(p≤0.001), ∼5.94−(p = 0.010) and ∼6.37-fold (p = 0.001) decrease in the cell growth at 24, 48 and 72 h, respectively, compared to the untreated control cells (Figure 1B). This was further supported by colony formation and soft agar assays wherein a dose-dependent decrease in the number of colonies was observed in both the cervical cancer cell lines (Figure 1C and D, respectively). Interestingly, at 80 µg/ml concentration, FR_aq_ significantly reduced the number of colonies in HeLa (∼4.97 fold; p≤0.001) and SiHa (∼2.95 fold; p≤0.001) compared to their respective untreated control cells (Figure 1D). Thus, Ficus regulated the growth kinetics of cervical cancer cells in a significant manner. As a negative control, we took C33A (HPV negative) cell line and analyzed the cytotoxicity of FR_aq_ in it. Ficus did not induce any cytotoxicity up to 160 µg/ml concentration in C33A cells, which was similar to that observed in SiHa and HeLa (Figure S1). However, at higher concentrations, FR_aq_ induced cytotoxicity in all the three cell lines, wherein HeLa and C33A cells showed similar cytotoxic effect.

### Ficus Induces G_1_ Phase Arrest in SiHa and Alters the Expression of Cell Cycle Regulatory Proteins

**Figure 2:**
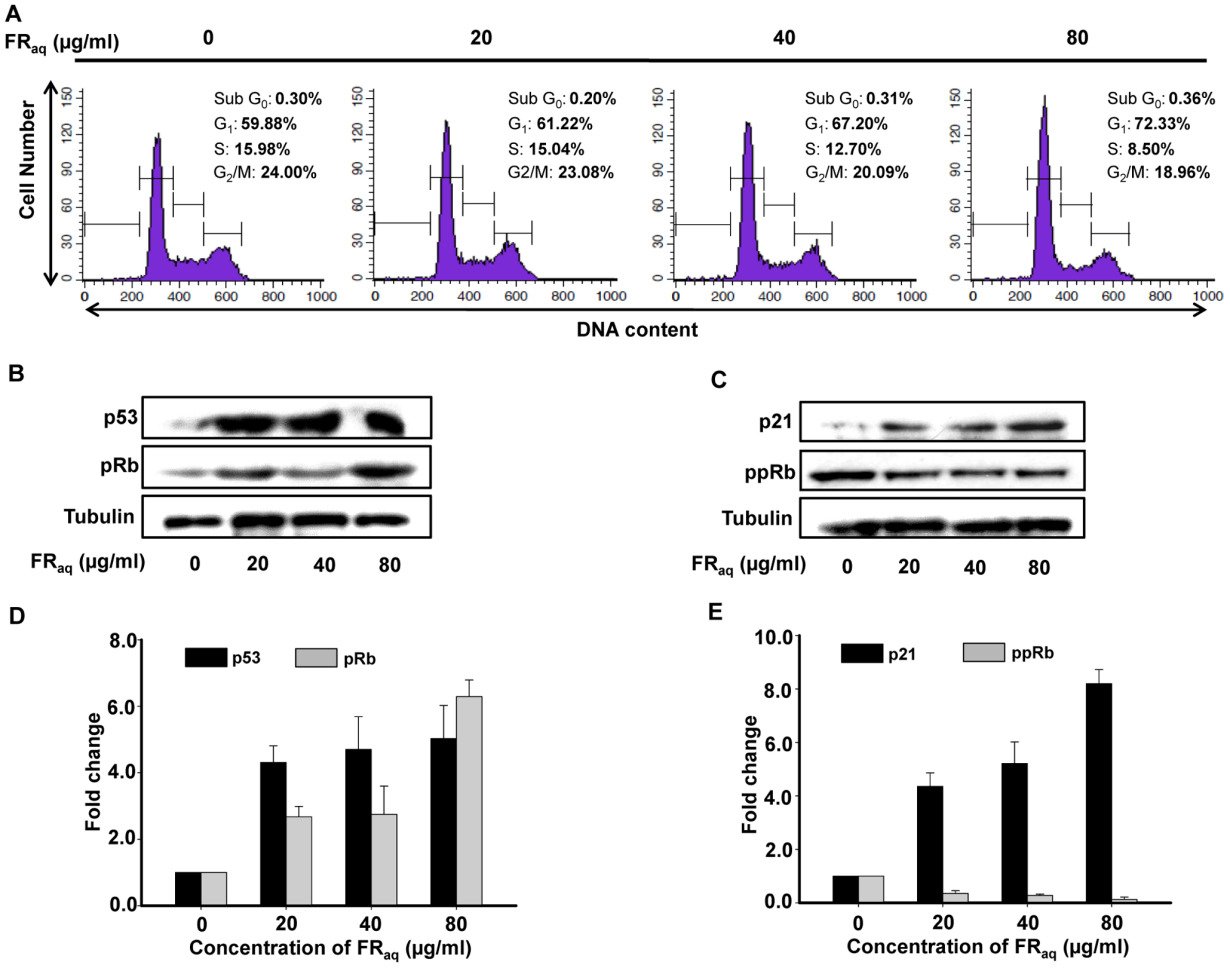
Ficus arrests the cell cycle in SiHa at G_1_/S phase and modulates the expression of cell cycle regulatory proteins. SiHa cells were treated with different concentrations of FR_aq_ (0–80 µg/ml) for 24 h. (**A**) Enhanced accumulation of the cells in G_1_ phase with a concomitant decrease in S-phase population was observed after treatment with Ficus (as indicated by histograms). Western blot shows the expression levels of p53 and pRb (**B**) as well as p21 and ppRb (**C**). Tubulin was used as a loading control. (**D, E**) Densitometric analysis of the western blot showing fold change in protein levels upon FR_aq_treatment. The bands were quantified by densitometry scanning using ImageJ 1.44p (Wayne Rasband, National Institutes of Health, USA, http://imagej.nih.gov/ij). The data represents mean ± SD of three independent experiments.

To analyze the mechanism behind the Ficus mediated regulation of growth kinetics in cervical cancer cells, we investigated the cell cycle distribution in SiHa, HeLa and C33A. Flow cytometry analysis showed that in presence of FR_aq_, SiHa exhibited an increase in G_1_ population with a simultaneous decrease in S phase in a dose-dependent manner (Figure 2A). Interestingly, at 80 µg/ml concentration, there was an increase in the percentage of cells in G_1_ phase (from 59.88 to 72.33%) with a simultaneous decrease in the S phase population (from 15.98 to 8.50%, p<0.050). On the other hand, in HeLa, there was a significant increase in sub-G_0_ population (from 3.65 to 87.38%, p<0.001) indicating apoptotic population (Figure S2). Interestingly, at non-toxic doses, FR_aq_ did not affect the growth of HPV negative C33A cells (Figure S2).

We investigated the mechanism of G_1_/S phase arrest in SiHa by evaluating the expression of G_1_ checkpoint proteins such as p53, pRb, phospho Rb (ppRb) and p21. There was a significant increase in the expression of p53 (Figure 2B and D) as well as its downstream effector, p21 (Figure 2C and E) after treatment of the cells with FR_aq_. The expression of pRb was analyzed since dephosphorylated pRb is known to form complexes with E2F to repress the transcription of cell proliferative genes [30]. FR_aq_, significantly increased the expression of pRb (Figure 2B and D) with a simultaneous decrease in the levels of ppRb (Figure 2C and E) in a dose-dependent manner. These results suggest that Ficus induced G_1_/S arrest in SiHa by modulating the expression of the cell cycle regulatory proteins.

### Ficus Induces Apoptosis in HeLa through Increase in Cyt c and Caspase 3 Expression

**Figure 3:**
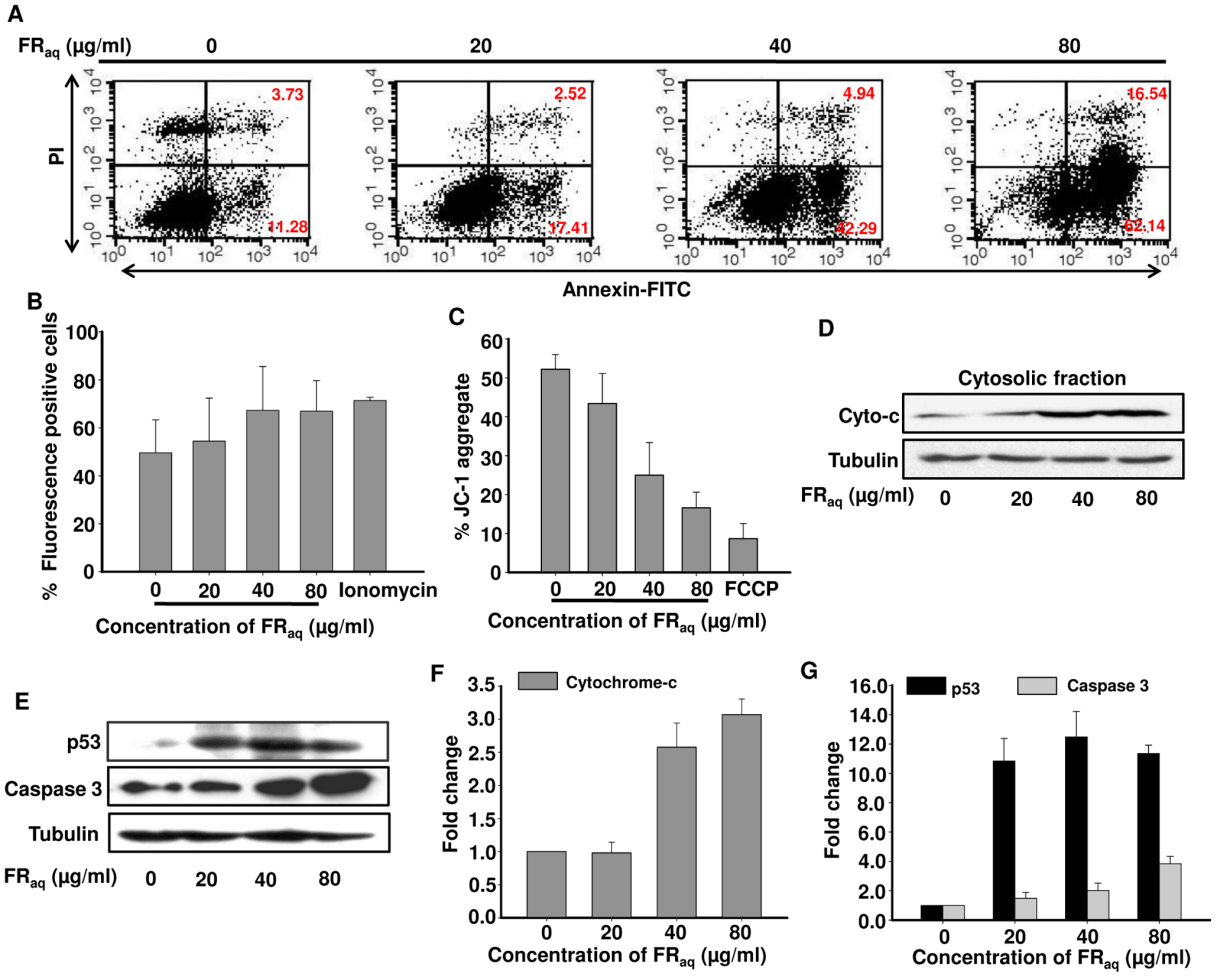
Ficus induces apoptosis in HeLa through mitochondrial dependent pathway. (**A**) Representative FACS pictograms of cells treated with FR**_aq_**(0–80 µg/ml) are shown. Percent of annexin V-positive (early-apoptotic cells, lower right quadrant) and Annexin V/PI-double-positive cells (late-apoptotic cells, upper right quadrant) are indicated. (**B**) Flow cytometric analysis of the rapid calcium release in HeLa cells after treatment with FR**_aq_**(0–80 µg/ml) has been shown. Ionomycin was used as a positive control. The data represents mean ± SD of three independent experiments. (**C**) FACS analysis following JC-1 staining of HeLa showed alteration of the mitochondrial membrane potential after FR**_aq_**(0–80 µg/ml) treatment compared to untreated control cells. The data represents mean ± SD of three independent experiments. (**D**) Western blot shows the expression of cytochrome c from cytosolic fraction. Tubulin was used as a loading control. (**E**) Total protein was isolated and analysed for expression of p53 and caspase 3 by immunoblotting. Tubulin was used as a loading control. (**F and G**) Densitometric analysis of the western blot showing fold change in protein levels. The bands were quantified by using ImageJ 1.44p (Wayne Rasband, National Institutes of Health, USA, http://imagej.nih.gov/ij).

We found that in HeLa, Ficus treatment resulted into increase in the number of cells in sub-G_0_ phase, indicative of apoptotic population (Figure S2). On staining with Annexin V-FITC, the cells showed a dose-dependent increase in both early as well as late apoptotic cell population (Figure 3A). Interestingly, at 80 µg/ml FR_aq_ concentration, there was ∼4.4-fold (p≤0.050) and ∼5.5-fold (p≤0.050) increase in both early as well as late apoptotic cell population, respectively, compared to the untreated control cells. On the other hand, no apoptosis was observed in FR_aq_ treated SiHa or C33A cells (Figure S3).

We studied Ca^2+^ signaling mechanism in cells treated with FR_aq_ and observed that it induced a dose-dependent increase in the intracellular calcium levels (Figure 3B). Ionomycin was used as a positive control. Interestingly, the increase in intracellular calcium resulted into disruption of the mitochondrial membrane potential (Δψm) that was observed by decrease in red fluorescence intensity, after staining the cells with JC-1 dye (Figure 3C). There was ∼3-fold reduction in the red fluorescence intensity (p≤0.001) at 80 µg/ml concentration of FR_aq._ FCCP was used as a positive control in the study. The mitochondrial membrane depolarization was associated with a dose-dependent increase in the cytosolic cytochrome c (Figure 3D and F) that was accompanied by an increase in the expression of caspase 3 and p53 (Figure 3E and G). These results indicate that Ficus induced apoptosis in HeLa through mitochondrial dependent pathway.

### Ficus Decreases Invasion and Migration of SiHa and HeLa

**Figure 4:**
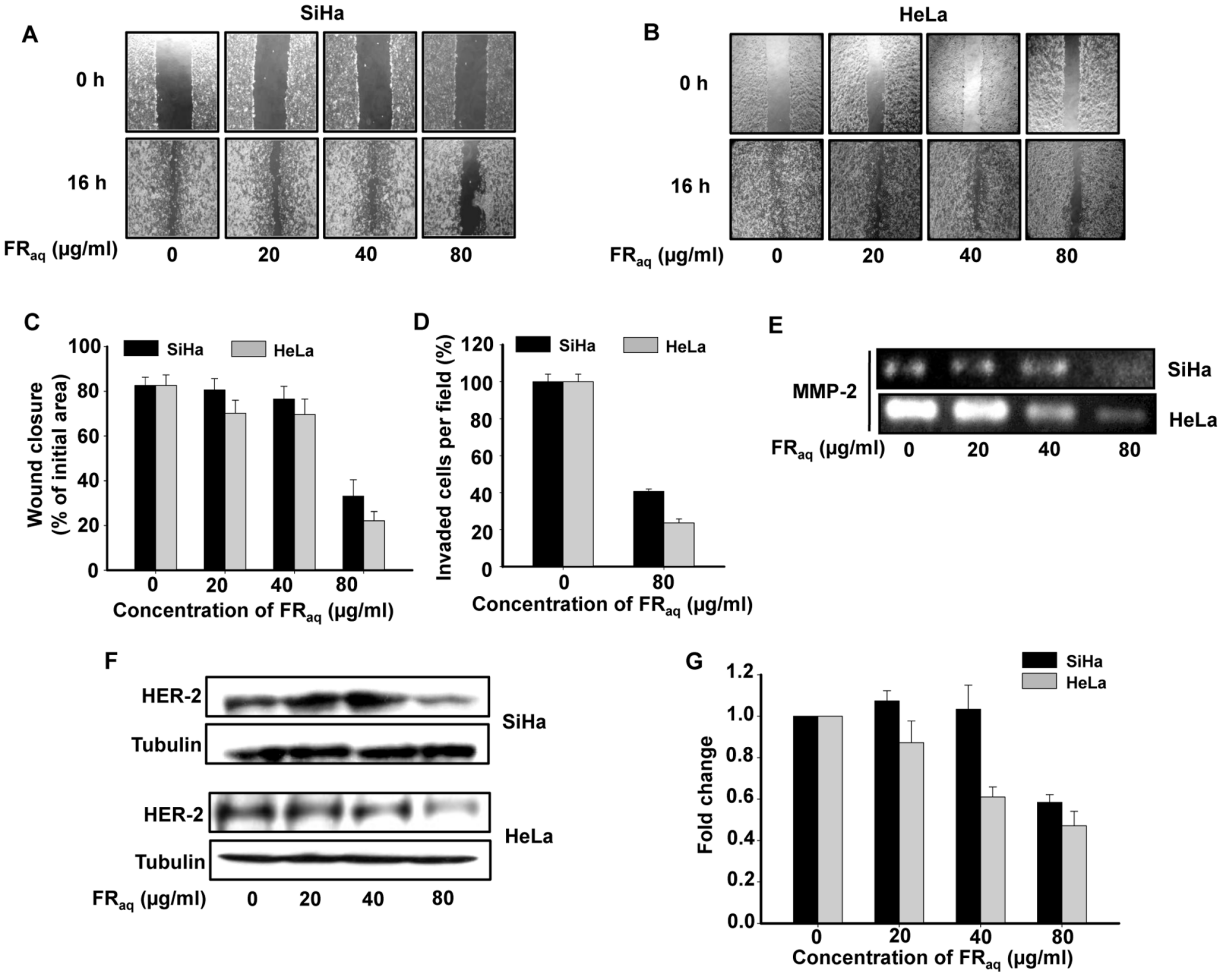
Ficus regulates invasion and migration of cervical cancer cells. Analysis of cell migration in SiHa (**A**) and HeLa (**B**) treated with FR_aq_ (0–80 µg/ml) was measured by wound-healing assay. The upper panel of the image shows the wound made at 0 h. The lower panel shows the migration of cells corresponding to the distance travelled at 16 h. (**C**) Graphical representation of wound closure in SiHa and HeLa cells at 16 h after FR_aq_ treatment has been shown. Values were represented as the percent wound closure and expressed as mean ± SD for three independent experiments. (**D**) Cell invasion assay showing the percentage of cells invaded per field in the presence or absence of FR**_aq_**. The invaded cells were counted in ten random fields and the values have been expressed as mean ± SD for three independent experiments. (**E**) Gelatin zymography showing downregulation of MMP-2 expression in FR**_aq_** (0–80 µg/ml) treated SiHa and HeLa. (**F**) Western blot analysis showing decrease in Her-2 expression in SiHa and HeLa treated with FR**_aq_** (0–80 µg/ml). Tubulin was used as a loading control. (**G**) Densitometric analysis of the western blot showing fold change in HER-2 protein levels in SiHa and HeLa. The bands were quantified by densitometry using ImageJ 1.44p (Wayne Rasband, National Institutes of Health, USA, http://imagej.nih.gov/ij).

Wound healing assay was performed in both the cell lines and it was observed that Ficus effectively inhibited the migration of both SiHa (Figure 4A) and HeLa (Figure 4B) in a dose- and time-dependent manner compared to the untreated control cells. After 16 h, the untreated SiHa and HeLa cells were able to cover up ∼82% of the wound, whereas at 80 µg/ml of FR_aq_ treatment, the cells covered up the wound by ∼33% (p<0.001) and 22% (p<0.001), respectively (Figure 4C). At this particular dose, Ficus reduced the invasive capability of both SiHa and HeLa by ∼2.45−(p≤0.001) and ∼3.8-folds (p≤0.001), respectively, compared to the untreated control cells (Figure 4D).

It is well known that increased expression of MMPs in tumor tissues is associated with cancer cell matrix degradation, invasion as well as metastasis [31]. We observed that FR_aq_ significantly down-regulated the expression of MMP-2 in both SiHa and HeLa cells (Figure 4E) compared to the untreated control cells.

HER2/*neu* has been reported to enhance the metastatic potential of cancers cells [32] and is positively correlated with MMP-2 expression [33]. We found that FR_aq_ decreased the expression of Her-2 in a dose-dependent manner in both SiHa and HeLa (Figure 4F and G). The data suggest that Ficus reduced the migration as well as invasion of cervical cancer cells by modulating the expression of Her-2 and MMP-2 proteins.

### Ficus Reduces the Expression of viral Oncoproteins E6 and E7

**Figure 5:**
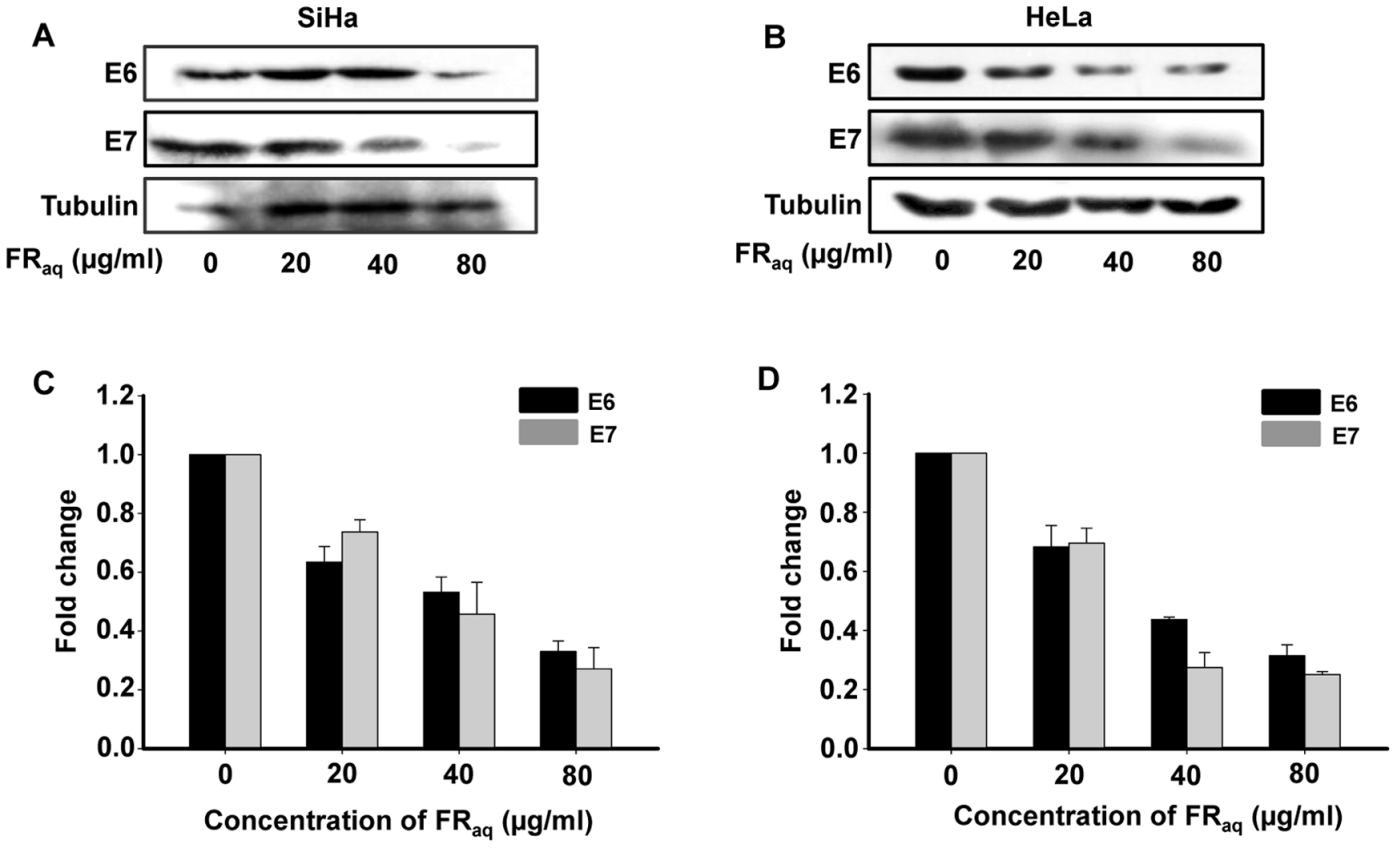
Ficus decreases the expression of E6 and E7 proteins. The expression of E6 and E7 oncoproteins was determined by immunoblotting with E6 and E7 antibodies in SiHa (**A**) and HeLa (**B**) treated with FR**_aq_** (0–80 µg/ml). Tubulin was used as a loading control. Densitometric analysis of the western blot showing fold change in E6 and E7 protein levels upon FR**_aq_** treatment in SiHa (**C**) and HeLa (**D**). The bands were quantified by densitometry using ImageJ 1.44p (Wayne Rasband, National Institutes of Health, USA, http://imagej.nih.gov/ij).

Since, Ficus exhibited significant antineoplastic potential in both HPV16 (SiHa) and HPV18 (HeLa) positive cell lines, we investigated the expression of the viral proteins E6 and E7 in the treated and untreated cells. It was observed that, FR_aq_ significantly reduced the expression of E6 and E7 oncoproteins in both SiHa and HeLa (Figure 5A and B, respectively). At 80 µg/ml FR_aq_ concentration, the expression of E6 and E7 proteins were decreased by ∼3.0−(p≤0.001) and 3.7-folds (p≤0.001), respectively, in SiHa (Figure 5A and C) and by ∼3.2−(p≤0.001) and 4.0-folds (p≤0.001), respectively, in HeLa compared to the untreated control cells (Figure 5B and D). Thus, Ficus decreased the expression of the viral oncoproteins E6 and E7, which potentiates its therapeutic significance in cancer regulation.

## Discussion

Cervical cancer is one of the most common cancers in the women worldwide [1], [34]. HPV infection is considered to be the main risk factor for the development of cervical cancer [35] wherein HPV-16 and -18 account for about 70% of the invasive cancers [3]. Although surgery and chemoradiotherapy can cure 80–95% of women with early-stage and 60% of loco-regionally advanced cancer, the recurrent and metastatic disease still remains a major concern [36]. Recently, lot of attention is being focused towards identification of new alternative approaches that would reduce morbidity as well as side-effects conferred by conventional chemotherapy. Currently, the plants, vegetables, herbs and spices used in folk and traditional medicine have been accepted as one of the main sources of chemopreventive drugs [8], [9].

*F. religiosa* has been extensively used in the management of various disorders [36]. Recently, the pro-apoptotic activity of *F. religiosa* leaf extract (acetone fraction) was reported in breast cancer cells [14]. We have previously reported that the aqueous and ethanolic extracts of *F. religiosa* bark exhibited significant ‘total antioxidant capacity’ and showed appreciable cytotoxicity in cervical cancer cell lines [22]. In the present study, we have further elucidated the anti-neoplastic potential of the aqueous extract of *F. religiosa* bark (FR_aq_) in cervical cancer cells with the possible underlying mechanisms. It was observed that FR_aq_ regulated the growth kinetics of the cervical cancer cells lines in a statistically significant manner and thus, Ficus exhibited a promising anticancer potential.

p53, a master tumor suppressor, is the most frequently mutated gene in almost all kinds of human cancers [37]. Moreover, loss of p53 function is responsible for the progression to more aggressive cancer phenotype [38]. In cervical cancer, E6 from high-risk HPV types (16 and 18) initiates degradation of p53 and thus, restoration of its function could be an effective therapeutic approach [39]. Reactivation of p53 in cervical cancer cells can lead to inhibition of cell proliferation as well as induction of apoptosis [40]. Most of the chemopreventive drugs regulate the growth of cancer cells either by arresting them at G_1_/S or G_2_/M phase or by induction of apoptosis by p53-dependent or independent mechanisms [41]. In our studies, we found that FR_aq_ exerted its anti-proliferative activity in each of the cervical cancer cell line by different mechanisms. In HPV-16 positive SiHa cells, FR_aq_ induced G_1_/S phase arrest through increase in the expression of p53 and p21 with a simultaneous decrease in the phosphorylation of pRb tumor suppressor protein. p21^WAF1/CIP1^, a cyclin-dependent kinase inhibitor, is a p53-inducible protein that blocks the cell cycle progression in the G_1_/S phase [42]. Thus, up-regulation of p21 by FR_aq_ might have resulted into activation of downstream effectors of p53-dependent G_1_/S arrest. The hypophosphorylated form of retinoblastoma protein (pRb), a tumor suppressor, forms a complex with E2F transcription factor resulting into repression of cell proliferative genes [43]. The viral E7 oncoprotein is known to inactivate the complex formation between pRb and E2F, thereby resulting into destabilization of pRB that eventually leads to deregulation of the cell cycle [44]. In our studies, we observed that Ficus reduced the levels of ppRb that might have resulted into increased expression of pRb as well as eventual arrest of cells in G_1_/S phase.

Apoptosis is an important mechanism to kill the tumor cells and [45] can be induced by increase in the mitochondrial calcium that results into loss of membrane potential (Δψm), expansion of the matrix and rupture of the outer mitochondrial membrane [46]. This results into release of cyt c into the cytosol, either by inhibition of anti-apoptotic factors or activation of pro-apoptotic proteins leading to the activation of caspase 3/9 [47]. During cell death, mitochondria are known to accumulate Ca^2+^, resulting into activation of the permeability transition pore (PTP) that leads to transient mitochondrial depolarization [48]. This leads to release of cyt c along with a large number of other factors from the inter-membrane space [49]. In HPV-18 positive HeLa cells, FR_aq_ induced p53-dependent apoptosis through increase in intracellular calcium and depolarization of mitochondrial membrane potential that lead to release of cytosolic cyt c and increase in caspase 3 expression. Interestingly, at non-cytotoxic dose, Ficus didn’t induce either arrest or apoptosis in C33a (HPV-negative, p53 mutated), thereby suggesting alternate mechanisms of cell death.

The observed dichotomy in the regulation of growth in SiHa and HeLa could be due to variation in p53 activation that may decide the fate of a cell to either initiate apoptosis or undergo cell cycle arrest. It has already been reported that low levels of p53 induce cell cycle arrest whereas high levels of p53 induce apoptosis [50], [51]. Our results show that p53 was activated more in HeLa compared to SiHa in response to FR_aq_ treatment, thereby, resulting into apoptosis in the former and cell cycle arrest in the latter. The other reason for the altered response of SiHa and HeLa towards Ficus treatment could be the difference in their genetic make-up that includes their HPV status as well as the viral copy number [52]. For example, SiHa (squamous cell carcinoma) contains around 1–2 integrated copies of HPV 16 genome whereas HeLa (adenocarcinoma) has around 10–50 integrated copies of HPV 18 [52], [53]. Moreover, the rate of replication is also different in both the cell types.

HER2/neu oncogene is frequently amplified in cervical cancer and can be considered as a potent therapeutic target [54], [55]. Its overexpression has been found to be associated with up-regulation of MMP-2 and MMP-9 that play an important role in cancer cell invasion and metastasis [33]. Interestingly, Ficus significantly reduced the expression of both HER-2 and MMP-2 that might have resulted in the observed decrease in the migration as well as invasion of cervical cancer cells.

E6 and E7 are the two viral oncoproteins known to induce cervical cancer by inactivating the tumor suppressor proteins, p53 and pRb, respectively [39]. p53 gene is mutated irreversibly in most of the cancers; however, cervical carcinomas and cell lines have been reported to retain wild-type p53 and pRb genes whose function gets masked by the viral E6 and E7 proteins [56]. We observed that Ficus decreased the expression of E6 and E7 in both the cervical cancer cell lines. The down regulation of E6 and E7 oncoproteins might have led to the restoration of tumor suppressor functions of p53 and pRb proteins, respectively. This might have led to the activation of downstream signaling molecules resulting into either cell cycle arrest or apoptosis. Even though a direct effect of E6/E7 on Her-2 has not been reported, however, their coexpression has been demonstrated to be critical for induction of head and neck squamous cell carcinomas (HPV positive) [57] as well as breast cancer [58]. These data suggest that *F. religiosa* has the potential to target HPV E6 and E7 proteins that could have a significant therapeutic potential in cervical cancer.

The “reverse pharmacology” or “bed to benchside” approach seems to be gaining importance in the current scenario of identifying new anticancer drugs with improved therapeutics and lesser or no side-effects [59]. This seems to be a viable approach to validate the traditional medicines for their possible drug development. Even though the modern drug design prefers using a single chemical entity with specific molecular targets, it may not be beneficial to the patient, partly, due to the possibility of development of resistance or associated side-effects. On the contrary, the whole plant extract may prove to be more efficacious due to presence of high content of polyphenols that may exhibit improved bioavailability and lower toxicity compared to the single chemical entity. *F. religiosa* is rich in polyphenols [36] and we have also confirmed the presence of flavonoids, phenols, saponins, tannins and carbohydrates in FR_aq_ (Table S1). Owing to their chemopreventive properties, polyphenols can modulate the process of carcinogenesis either towards protective or therapeutic side depending upon either the amount of the drug being used or upon the cellular phenotype [60]. Thus, our findings provide a strong basis for further exploration of *F religiosa* as a therapeutic drug against cervical cancer, either alone or as an adjuvant to standard chemotherapeutic agents.

## Supporting Information

Figure S1 **Cytotoxic effect of FR_aq_ in cervical cancer cell lines.** SiHa, HeLa and C33A were treated with different concentrations (0–620 µg/ml) of FR_aq_ for 24 h. The viability was measured by MTT assay.
(TIF)

Figure S2 **Effect of Ficus on cell cycle in HeLa and C33A.** Cervical cancer cell lines HeLa and C33A, were treated with different concentrations of FR**_aq_** (0–80 µg/ml) for 24 h. Distribution of cells in different phases of cell cycle was analyzed by propidium iodide (PI) staining followed by flow cytometry. Increase in HeLa cell population in sub G_0_ phase, was indicative of apoptosis whereas there was no change in cell cycle profile in HPV negative C33A cells, upon treatment with FR**_aq_**.
(TIF)

Figure S3 **Effect of Ficus on apoptosis in SiHa and C33A.** Representative FACS pictograms of SiHa and C33A cells treated with FR**_aq_** (0–80 µg/ml) for 24 h are shown that have been analyzed for apoptosis by Annexin V/PI staining. The lower left quadrants of each panels show the viable cells (negative for both PI and Annexin V-FITC). The upper right quadrants contain late apoptotic cells (positive for both PI and Annexin V-FITC). The lower right quadrants represent the early apoptotic cells (Annexin V-FITC positive and PI negative).
(TIF)

Table S1 **Preliminary phytochemical analysis of FR_aq_.**
(TIF)
