# Supplementary figures and images for: The Aqueous Extract of Ficus religiosa Induces Cell Cycle Arrest in Human Cervical Cancer Cell Lines SiHa (HPV-16 Positive) and Apoptosis in HeLa (HPV-18 Positive)
Source: PLoS One. 2013 Jul 26;8(7):e70127. doi: 10.1371/journal.pone.0070127 (PMC3724825; doi:10.1371/journal.pone.0070127)

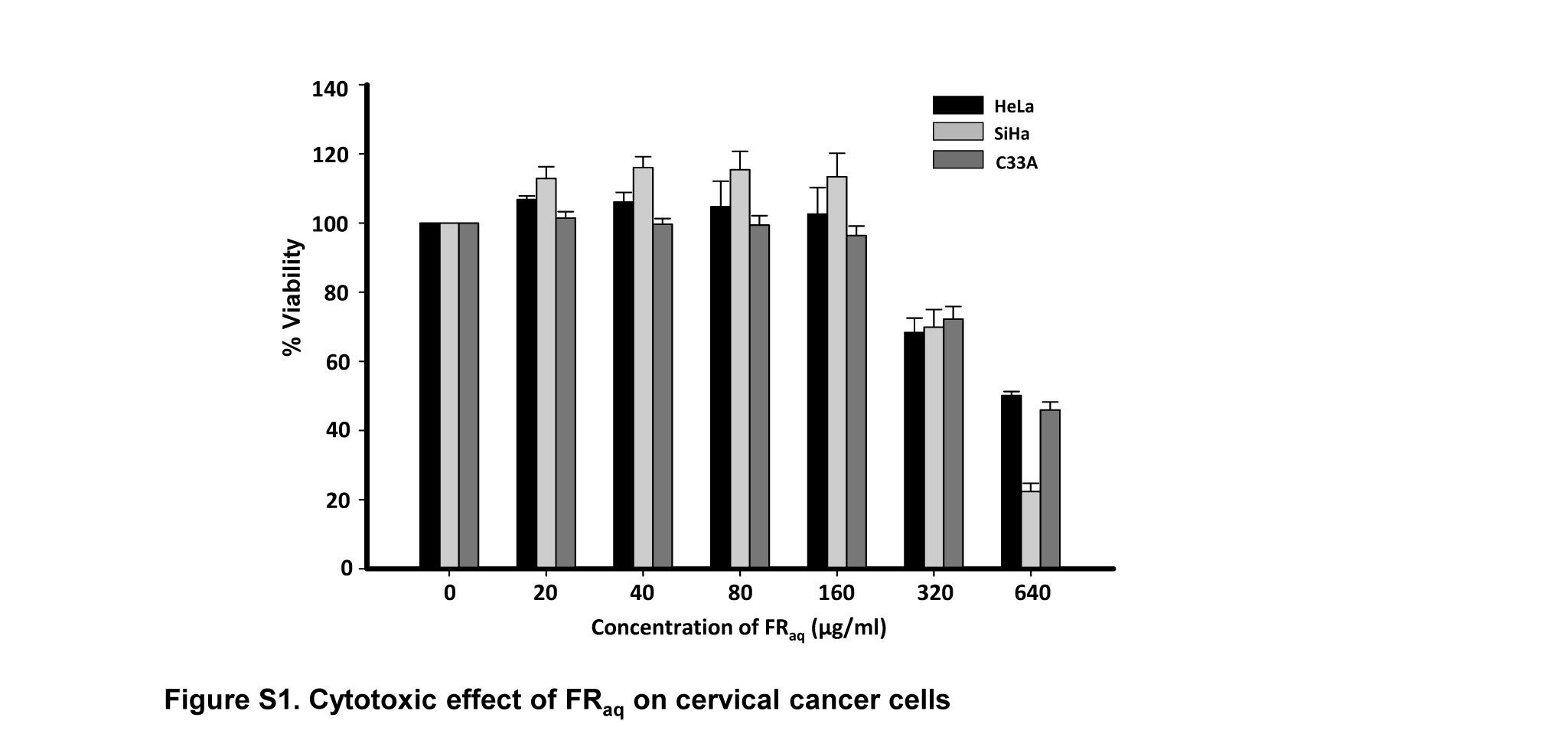

Supplement: Figure S1 — Cytotoxic effect of FRaq in cervical cancer cell lines. SiHa, HeLa and C33A were treated with different concentrations (0–620 µg/ml) of FRaq for 24 h. The viability was measured by MTT assay. (TIF) [file pone.0070127.s001.tif]

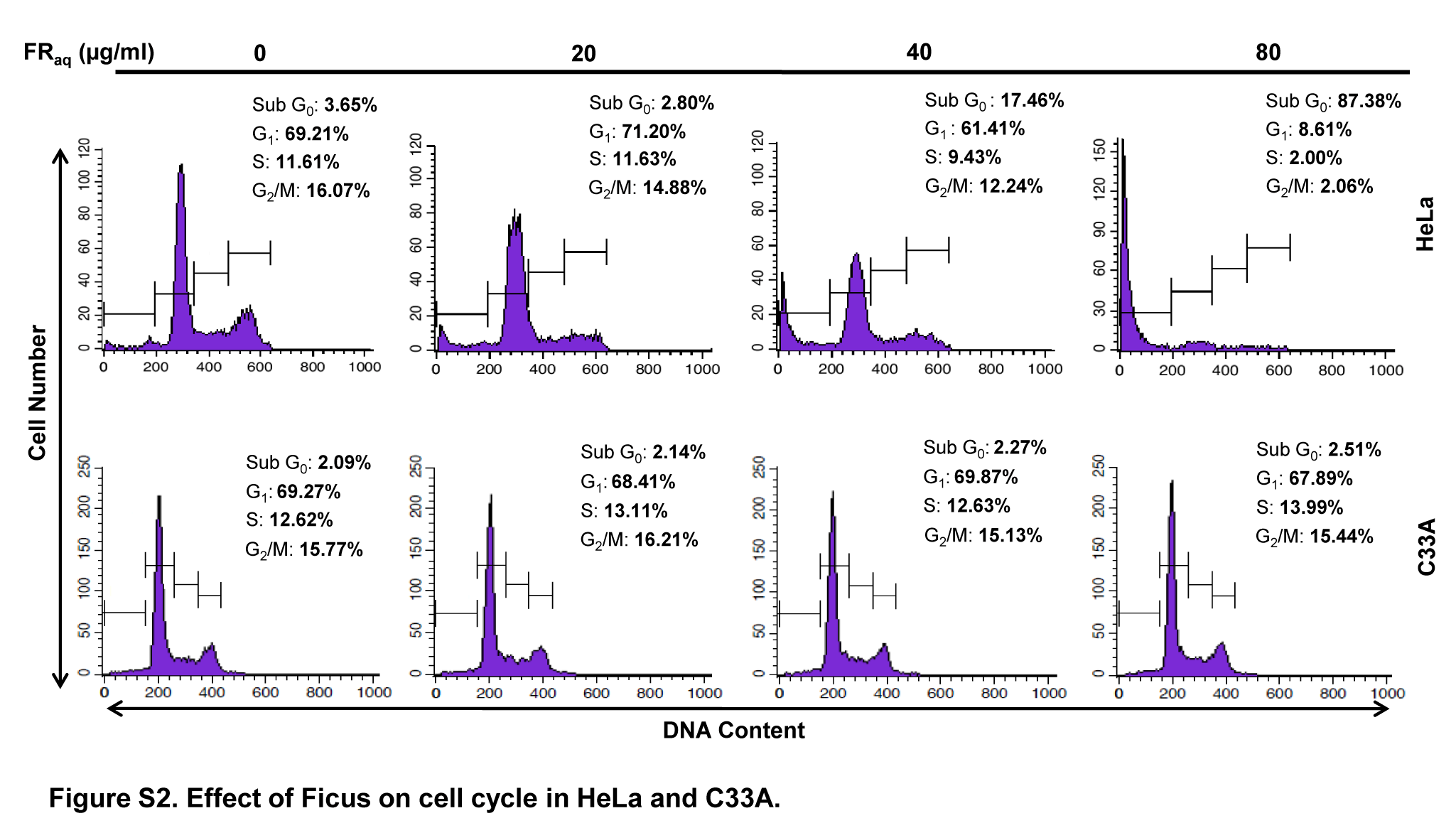

Supplement: Figure S2 — Effect of Ficus on cell cycle in HeLa and C33A. Cervical cancer cell lines HeLa and C33A, were treated with different concentrations of FRaq (0–80 µg/ml) for 24 h. Distribution of cells in different phases of cell cycle was analyzed by propidium iodide (PI) staining followed by flow cytometry. Increase in HeLa cell population in sub G0 phase, was indicative of apoptosis whereas there was no change in cell cycle profile in HPV negative C33A cells, upon treatment with FRaq. (TIF) [file pone.0070127.s002.tif]

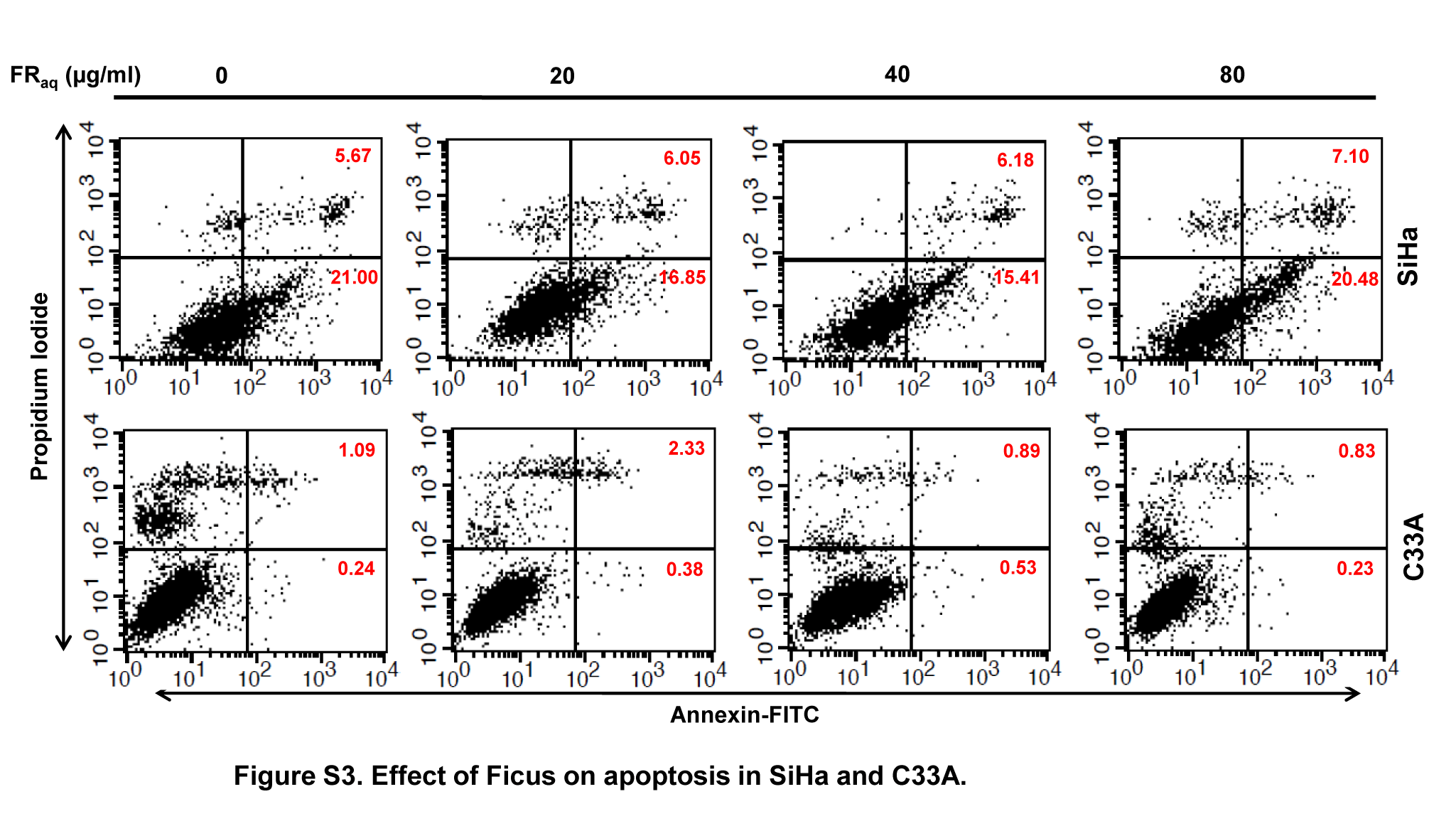

Supplement: Figure S3 — Effect of Ficus on apoptosis in SiHa and C33A. Representative FACS pictograms of SiHa and C33A cells treated with FRaq (0–80 µg/ml) for 24 h are shown that have been analyzed for apoptosis by Annexin V/PI staining. The lower left quadrants of each panels show the viable cells (negative for both PI and Annexin V-FITC). The upper right quadrants contain late apoptotic cells (positive for both PI and Annexin V-FITC). The lower right quadrants represent the early apoptotic cells (Annexin V-FITC positive and PI negative). (TIF) [file pone.0070127.s003.tif]

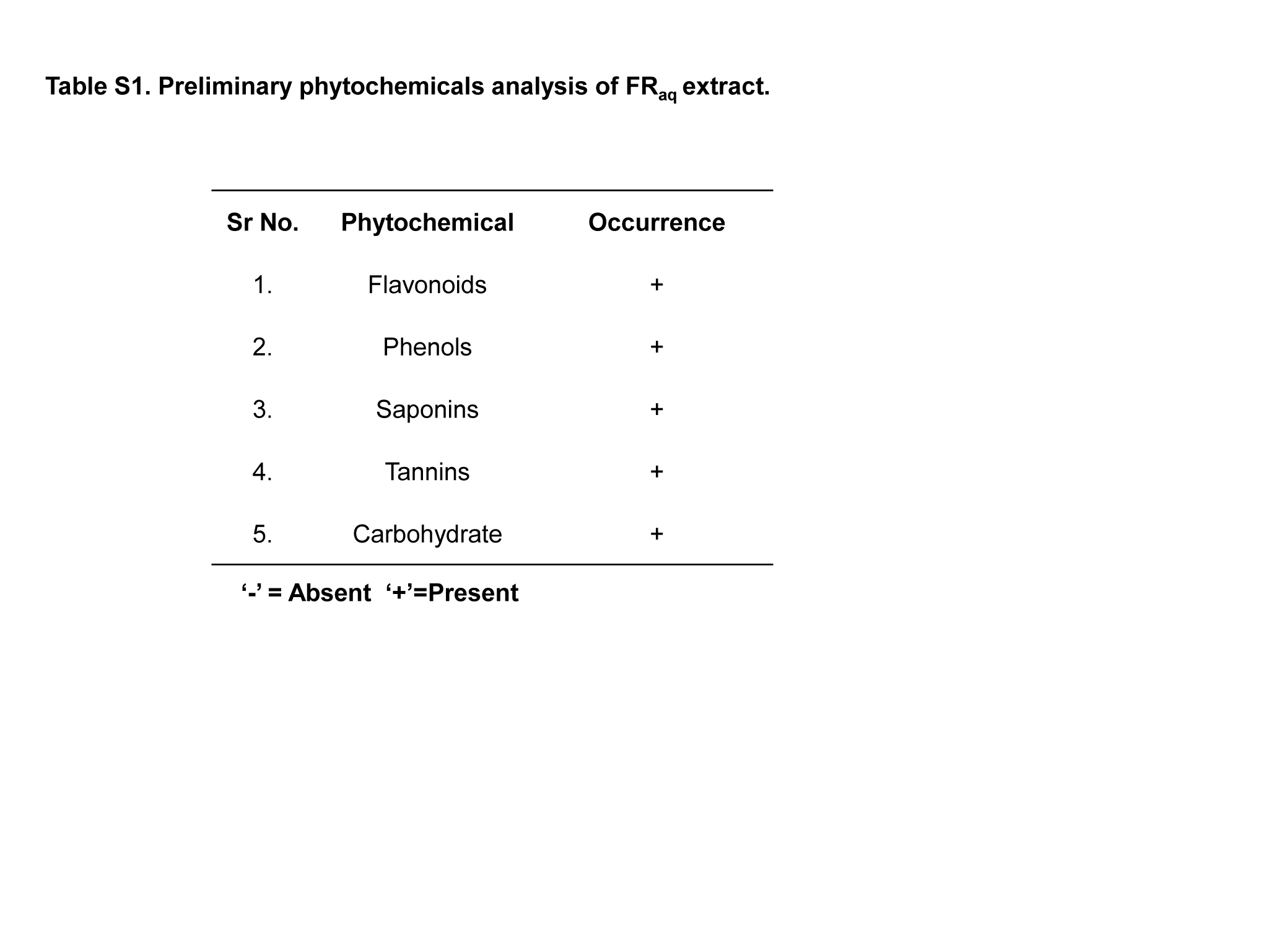

Supplement: Table S1 — Preliminary phytochemical analysis of FRaq. (TIF) [file pone.0070127.s004.tif]
